# A population-based study on hyperinsulinaemia and arterial stiffness in men with and without abdominal aortic aneurysm

**DOI:** 10.1097/XCE.0000000000000290

**Published:** 2023-09-18

**Authors:** Shahab Fatemi, Stefan Acosta, Moncef Zarrouk, Peter M. Nilsson, Anders Gottsäter

**Affiliations:** a Department of Clinical Sciences, Lund University, Malmö; b Department of Medicine, Trelleborg Hospital; Departments of c Cardiothoracic and Vascular Surgery; d Internal and Emergency Medicine, Skåne University Hospital, Malmö, Sweden

**Keywords:** abdominal aortic aneurysm, aortic diameter, arterial stiffness, atherosclerosis, diabetes mellitus, hyperinsulinaemia, metabolic syndrome, screening

## Abstract

**Objectives:**

Patients with type 2 diabetes mellitus (DM) run lower risk for abdominal aortic aneurysm (AAA, aortic diameter ≥ 30 mm) and its complications. We aimed to evaluate associations between disturbances in glucose metabolism and arterial stiffness, AAA, and abdominal aortic diameter in 65-year-old men.

**Methods:**

Forty-eight 65-year-old men with screening-detected AAA and 115 men with normal abdominal aortic diameter underwent examination of glucose metabolism and arterial stiffness.

**Results:**

Men with AAA had higher BMI, waist-hip ratio (WHR), frequency of DM, haemoglobin A_1c_, smoking exposure, and plasma insulin levels at 0, 60 and 120 min during OGTT compared to those without. The increase in p-insulin (*P* < 0.001) after OGTT was also higher in men with AAA, adjusted for smoking, WHR, and nadir value of p-insulin. In analyses adjusted for smoking, use of lipid-lowering agents, and WHR, the increase in p-insulin at 2-hours (*P* = 0.006) after OGTT and p-homocysteine were associated with abdominal aortic diameter. There were no differences between groups in aortic stiffness or skin autofluorescence Advanced Glycation End products.

**Conclusion:**

In this population-based study hyperinsulinaemia as a marker of insulin resistance, but not hyperglycaemia or aortic stiffness, was associated with AAA and abdominal aortic diameter in 65-year-old men.

## Introduction

Type 2 diabetes mellitus (DM) affects nearly 350 million people worldwide [1–3], causing both systemic atherosclerosis and microvascular complications through effects upon platelet, endothelial cell, and smooth muscle cell function [4–7]. These effects make type 2 diabetes one of the leading risk factors for atherosclerotic vascular disease [8].

The most common manifestation of aneurysmal disease, abdominal aortic aneurysm (AAA, abdominal aortic diameter ≥30 mm), mainly affects men and is a potentially life-threatening condition due to the risk for aneurysm rupture [9]. Risk factors for AAA are partly the same as for atherosclerotic disease in general [10–12]. Inflammatory mediators, endothelial function, and the coagulation system interact with genetics and classic risk factors (age, hypertension, hyperlipidaemia, and smoking) in the pathogenesis of both conditions [10–12]).

Therefore, type 2 diabetes might also have been expected to be implicated in the process of aortic dilatation. Whereas the prevalence of type 2 diabetes is rising steeply due mostly to ageing populations [1], rates of AAA are, however, declining [13]. This could primarily be attributed to decreasing smoking rates [13], but the opposing trends could also indicate that patients with type 2 diabetes have a better chance of avoiding AAA and its complications [14]. Over time, a number of studies have confirmed this paradoxical association [14–17]. Several features of type 2 diabetes have been proposed to explain such potential protective effects; glycation of collagen and other arterial wall proteins [18], increased arterial stiffness and vascular ageing measured as carotid-femoral pulse wave velocity (c-f PWV) [19,20], renal diabetic complications leading to vascular calcification and medial sclerosis [21], or downregulation of matrix metalloproteinases [22] and chymases.

The risk factors for aortic aneurysmal disease are partly the same as for increased arterial stiffness; age, obesity, dyslipidemia, and arterial hypertension are implicated in both conditions [23,24]. Furthermore, the structural damage to the aortic wall in AAA might also directly influence its elasticity and compliance [25].

The aim of this exploratory study was to evaluate associations between disturbances in glucose metabolism and arterial stiffness, AAA, and abdominal aortic diameter in 65-year-old men.

## Material

All 65-year-old men from the city of Malmö and 15 neighbouring municipalities are invited to screening for AAA with ultrasound at the Department of Vascular Diseases, Skåne University Hospital, Malmö [26,27]. From 2010 to 2017, a total of 415 (1.7%) of 24 589 examined men were found to have AAA, and offered regular ultrasound examinations and prescription of statins. Out of a subgroup of 193 men undergoing examination and blood sampling [28,29], all 48 men consented to extended examination of glucose homeostasis and aortic stiffness. As a control group, we examined 115 screened 65-year-old men with aortic diameter <30 mm, matched for dates of examination and blood sampling.

## Methods

Weight (kg), height (m), and waist and hip circumferences (cm) were measured for calculation of BMI and waist-hip ratio (WHR).

Blood samples were centrifuged at 4^◦^C. Routine laboratory markers were analysed at the Department of Clinical Chemistry, Skåne University Hospital, Malmö (SWEDAC approved according to European norm 45001).

Plasma (p-) triglycerides, low-density lipoprotein, and high-density lipoprotein cholesterol levels were obtained utilising a Cobas device (Roche, USA).

Fasting p-glucose was analysed via biochromatic analysis (Cobas) [30]. Glycated haemoglobin A_1c_ (HbA_1c_) was calculated through capillary electrophoresis (Capillarys 3 Tera, Sebia, France). Glomerular filtration rate was calculated from p-creatinine, sex, and age and expressed in ml/min/1.73 m^2^. During oral glucose tolerance test (OGTT; 75 g glucose), p-glucose, serum (s-) insulin, and p-glucagon were analysed at 0, 60, and 120 min. The Hemocue Glucose System (HemoCue AB, Ängelholm, Sweden) was used to analyse p-glucose and the DakoELISA kit (Glostrup, Denmark) to analyse s-insulin (minimum detection level 3 pmol/l, intra- and interassay coefficients of variation 5.1–7.5% and 4.2–9.3%, respectively). P-glucagon was assayed with RIA GL-32K (Merck Millipore, Darmstadt, Germany, minimum detection level 18.5 pg/ml, intra- and interassay coefficients of variation 3.6–6.2% and 8.7–14.7%, respectively).

Aortic ultrasound examinations were performed using the LOGICe (General Electric Healthcare Inc, Chalfont S. Giles, UK). The maximal infrarenal anteroposterior diameter of the aorta was evaluated, and an AAA was defined as an aortic diameter of ≥30 mm, from the leading edge of the adventitia in the anterior wall to the leading edge of the intima of the posterior wall [31].

c-f PWV was measured using arterial tonometry [32] as the median of two measurements in supine position with a Sphygmocor (Atcor, Australia). Pulses were measured at the femoral artery and the carotid artery, and the distance between the two points multiplied by 0.8. The pulse transit time from carotid to femoral artery was divided by this length to estimate c-f PWV [33].

Skin autofluorescence (SAF) of Advanced Glycation End (AGE) products was estimated with an AGE Reader (DiagnOptics, Groningen, Netherlands) [34].

Systolic and diastolic brachial blood pressures were measured with an Omron device (Omron, USA) after 5 minutes rest, as the average of two measurements with 1-minute intervals. Ankle blood pressures were measured bilaterally. Central systolic blood pressure values were obtained from the c-f PWV analysis with the Sphygmocor.

### Definitions

DM was defined as fasting p-glucose ≥7.0 mmol/L or haemoglobin A1c ≥48 mmol/mol or 2-hour p-glucose ≥11 mmol/L. Impaired glucose tolerance was defined as fasting p-glucose < 7 mmol/L and 2-hour p-glucose ≥ 7.8 to < 11.0 mmol/L. Impaired fasting glycaemia was defined as fasting p-glucose 5.6 – 6.9 mmol/L and 2-hour p-glucose < 7.8 mmol/L [35]. Homeostatic Model Assessment for Insulin Resistance (HOMA-IR) was defined as (fasting p-glucose in mmol/L × fasting p-insulin in mU/L)/22.5.

Smoking was defined as current or former smoker.

Hypertension was defined as self-reported use of antihypertensive medication or blood pressure ≥140/90 mmHg. Peripheral arterial disease was defined as ankle-brachial index ≤ 0.90 [36] in at least one of the four ankle arteries.

### Ethical approval

All subjects gave written consent to participation, the study was approved by the Ethics Committee of Lund University (2010/239 and 2014/643), and all procedures were conducted in accordance with the Declaration of Helsinki.

### Statistics

Nominal data was expressed in percentages and differences between groups evaluated with Chi-square test. Continuous data was expressed with mean and SD, and group differences evaluated with independent samples t-test. Paired samples t-test was used to analyse the change in laboratory data between two different time points after OGTT. The mean change in p-glucose/s-insulin/p-glucagon from nadir value to 2-hour test was compared in men with and without AAA using univariate analysis of variance (ANOVA), adjusting for smoking, WHR, and nadir value of p-glucose, s-insulin, or p-glucagon, respectively. Association between hyperglycaemia variables and odds for AAA were evaluated with multi-variable logistic regression analysis, adjusted for smoking, WHR, and each hyperglycaemia variable separately, and odds were expressed as odds ratios (OR) with 95% confidence intervals (CI). Associations between plasma lipids and AAA presence, adjusted for treatment with lipid-lowering agents, were evaluated with logistic regression. Associations between laboratory variables and abdominal aortic diameter, adjusted for smoking, treatment with lipid-lowering agents, and WHR were evaluated with linear regression analysis. All *P*-values < 0.05 were considered statistically significant. Statistical analyses were performed using SPSS versions 27 and 28 (IBM, Armonk, New York, USA).

## Results

### Comparison of characteristics between men with and without AAA

Men with AAA had higher BMI, abdominal height, WHR, smoking exposure, and more often hypertension, peripheral arterial disease, and lipid-lowering treatment than those without AAA (Table 1). Total cholesterol, LDL-, and HDL cholesterol were lower in men with AAA despite adjustment for their higher use of lipid-lowering agents, whereas triglycerides, leukocytes, and homocysteine were higher in men with AAA compared to those with normal aortic diameter (Table 1). There were no differences between groups in blood pressure, c-f PWV, or SAF of AGE (Table 1).

**Table 1:** Background characteristics in 65-year-old men with and without abdominal aortic aneurysm (AAA) at ultrasound screening. N (%) or mean (SD)

	AAA (n = 48)	No AAA (n = 115)	*P*-value
Abdominal aortic diameter (mm)	37.8 (8.1)	19.5 (2.7)	NA
DM in first-degree relatives	13/46 (28.3)	29/111 (26.1)	0.78
BMI (kg/m^2^)	28.0 (3.2)	26.7 (3.7)	**0.035**
Abdominal height (cm)	24.3 (2.9)	22.2 (3.5)	**<0.001**
Waist-hip ratio	0.99 (0.05)	0.95 (0.07)	**<0.001**
Smoker/ex-smoker	37/44 (84.1)	61/106 (57.7)	**0.002**
Comorbidities			
Hypertension	41 (85.4)	69 (60.0)	**0.002**
Peripheral arterial disease	15 (31.2)	2 (1.7)	**<0.001**
Medication			
Antihypertensive	32 (66.7)	39/114 (34.2)	**<0.001**
Lipid-lowering	34 (70.8)	23/114 (20.2)	**<0.001**
Metformin	2 (4.2)	3/114 (2.6)	**0.63**
Blood pressures (mmHg)			
Systolic	141.1 (15.8)	143.0 (18.4)	0.56
Diastolic	83.4 (9.4)	83.5 (10.5)	0.95
Laboratory data			
P-total cholesterol (mmol/L)	4.1 (1.0) (n = 46)	5.1 (1.1)	**0.028** a
P-HDL cholesterol (mmol/L)	1.2 (0.3) (n = 46)	1.4 (0.4)	**0.019** a
P-LDL cholesterol (mmol/L)	2.6 (0.9) (n = 46)	3.4 (1.0)	**0.023** a
S-triglycerides (mmol/L)	1.8 (1.2) (n = 46)	1.4 (0.8)	**0.030** a
B-haemoglobin (g/L)	147 (13) (n = 46)	149 (9)	0.54
B-leukocytes (×10^9^/L)	7.2 (1.6) (n = 46)	6.1 (1.6)	<0.001
B-thrombocytes ((× 10^9^/L)	230 (63) (n = 46)	226 (38) (n = 113)	0.55
P-homocysteine (µmol/L)	20 (7) (n = 46)	17 (4) (n = 111)	**0.001**
Glomerular filtration rate (ml/min/1.73 m^2^)	67 (14) (n = 43)	71 (8) (n = 112)	0.078
Arterial tonometry			
c-f PWV (m/s)	9.5 (1.6) (n = 44)	9.3 (1.4) (n = 113)	0.55
Aortic Aix adjusted for heart rate 75/min	24.0 (12.5) (n = 45)	24.2 (10.3) (n = 114)	0.89
Central systolic blood pressure (mmHg)	123.7 (13.0) (n = 45)	120.8 (11.3) (n = 114)	0.16
Central diastolic blood pressure (mmHg)	78.9 (10.6) (n = 45)	78.8 (8.3) (n = 45)	0.95
Skin autofluorescence			
AGE (arbitrary units)	2.4 (0.5) (n = 45)	2.3 (0.5) (n = 112)	0.35

Bold indicates statistical significance of *P* values.

AAA, abdominal aortic aneurysm; AGE, advanced glycation end products; Aix, Augmentation index; B, blood; c-f PWV, carotid-femoral pulse wave velocity; DM, diabetes mellitus; HDL, high-density lipoprotein; LDL, low-density lipoprotein; NA, not applicable; P, plasma; S, serum.

a Adjusted for treatment with lipid-lowering agents.

### Comparison of glucose metabolism in men with and without AAA

Both the frequency of DM, mean haemoglobin A_1c_ values, and HOMA-IR were higher in men with screening-detected AAA compared to those without AAA (Table 2). During OGTT, s-insulin levels were higher at 0, 60, and 120 min in men with AAA compared to those without (Table 2). There was also a significantly higher increase in s-insulin in men with AAA compared to those with normal aortic diameter after OGTT, adjusted for smoking, WHR, and nadir value of s-insulin (Table 3).

**Table 2:** Glucose homeostasis in 65-year-old men with and without abdominal aortic aneurysm (AAA) at ultrasound screening. N (%) or mean (SD)

	AAA (n = 48)	No AAA (n = 115)	*P*-value
Diabetes mellitus	12/47 (25.5)	13/115 (11.3)	**0.023**
Impaired glucose tolerance	9/46 (19.6)	18/111 (16.2)	0.61
Impaired fasting glycaemia	17/47 (36.2)	48/113 (42.5)	0.46
Pre-diabetes or diabetes	36/47 (76.6)	78/113 (69.0)	0.34
Haemoglobin A1c (mmol/mol)	39.8 (10.0)	36.5 (4.5)	**0.005**
HOMA-IR	0.64 (0.38) (n = 42)	0.43 (0.28) (n = 113)	**<0.001**
Oral glucose tolerance test			
P-glucose (mmol/l, 0 min)	6.0 (0.9) (n = 43)	5.9 (0.8) (n = 113)	0.45
P-glucose (mmol/l, 60 min)	10.3 (2.7) (n = 43)	9.5 (2.3) (n = 112)	0.084
P-glucose (mmol/l, 120 min)	7.6 (2.7) (n = 43)	7.0 (2.3) (n = 112)	0.13
S-insulin (pmol/l, 0 min)	16.0 (8.7) (n = 42)	11.4 (6.8) (n = 114)	**<0.001**
S-insulin (pmol/l, 60 min)	117.2 (66.4) (n = 42)	83.7 (52.0) (n = 112)	**0.001**
S-insulin (pmol/l, 120 min)	90.5 (64.0) (n = 41)	57.3 (45.7) (n = 112)	**<0.001**
P-glucagon (pg/ml, 0 min)	43.0 (14.7)	40.2 (16.9)	0.36
P-glucagon (pg/ml, 120 min)	36.6 (11.6) (n = 39)	33.9 (14.0)	0.28

Bold indicates statistical significance of *P* values.

AAA, abdominal aortic aneurysm; HOMA-IR, homeostatic model assessment for insulin resistance; P, plasma; S, serum.

**Table 3:** Changes in p-glucose, s-insulin, and p-glucagon between nadir and 2-hours sample after oral glucose tolerance testing in 65-year-old men with and without abdominal aortic aneurysm (AAA) at ultrasound screening. Mean (SD) or mean (95% CI) as indicated

	Nadir mean (SD)	2-hours mean (SD)	Change mean (95% CI)	*P*-value
P-glucose (mmol/L)				
With AAA	6.0 (0.9)	7.6 (2.7)	1.6 (0.9–2.4)	**<0.001**
Without AAA	5.9 (0.8)	7.0 (2.3)	1.1 (0.8–1.4)	**<0.001**
aDifference in change in p-glucose between men with and without AAA			0.5 (−0.4 to 1.4)	0.28
S-insulin (pmol/L)				
With AAA	16.0 (8.7)	90.5 (64.0)	74.4 (56.1–92.7)	**<0.001**
Without AAA	11.4 (6.8)	57.3 (45.7)	46.1 (38.2–53.9)	**<0.001**
aDifference in increase in s-insulin between men with and without AAA			36.0 (23.4–48.6)	**<0.001**
P-glucagon (pg/ml)				
With AAA	43.0 (14.7)	36.6 (11.6)	−7.2 (−9.7 to −4.7)	**<0.001**
Without AAA	40.2 (16.9)	33.9 (14.0)	−6.3 (−7.7 to −4.8)	**<0.001**
aDifference in change in p-glucagon between men with and without AAA			−0.9 (−4.0 to 2.2)	**0.55**

Bold indicates statistical significance of *P* values.

CI, confidence interval; P, plasma; S, serum.

a Univariate analysis of variance (general linear model – univariate; adjusted for smoking, waist-hip ratio and nadir values of p-glucose, s-insulin, and p-glucagon.

### Glucose metabolism and presence of AAA

There were no associations between any variable reflecting hyperglycaemia and the odds for AAA, when adjusted for smoking and WHR (Table 4). When entering DM or pre-diabetes, smoking, and WHR as covariates, only smoking (OR 3.3, 95% CI 1.3 – 8.3; *P* = 0.013) and WHR (OR 4.2, 95% CI 1.7 – 10.2; *P* = 0.002) were independently associated with AAA.

**Table 4:** Associations between hyperglycaemic conditions and abdominal aortic aneurysm (AAA) in 65-year-old men undergoing ultrasound screening for AAA

Hyperglycaemic condition	Multi-variable regression	
	OR (95% CI)	*P*-value
Diabetes mellitus	1.8 (0.7–5.0)	0.25
Impaired glucose tolerance	1.0 (0.4–2.7)	0.99
Impaired fasting glycaemia	0.7 (0.3–1.6)	0.45
Diabetes mellitus or pre-diabetes	0.9 (0.4–2.1)	0.76

Adjusted for smoking, waist-hip ratio, and the respective hyperglycaemic conditions.

CI, confidence interval; OR, odds ratio.

### Glucose metabolism and abdominal aortic diameter

In analyses adjusted for smoking habits, lipid-lowering treatment, and WHR, the increase in s-insulin at 2 hours (*P* = 0.006) after OGTT and p-homocysteine (*P* = 0.033) were associated with abdominal aortic diameter, whereas there were no associations between any variable reflecting hyperglycaemia and abdominal aortic diameter (Table 5). Neither was aortic diameter associated with c-f PWV in adjusted analysis. When entering smoking and WHR in the linear regression model, WHR (*P* < 0.001) and smoking (*P* = 0.042) were both independently associated with abdominal aortic diameter.

**Table 5:** Linear regression model showing associations between laboratory variables and abdominal aortic diameter, adjusted for smoking, treatment with lipid-lowering agents, and waist-hip ratio in 65-year-old men undergoing ultrasound screening for abdominal aortic aneurysm

Variables	Standardized beta coefficient	*P*-value	B	95% CI for B
Fasting p-glucose	0.007	0.93	0.08	-1.7–1.8
Fasting s-insulin	0.10	0.20	0.13	-0.1–0.3
Increase in s-insulin at 2-hours after OGTT	0.22	**0.006**	0.04	0.01–0.08
Haemoglobin A1c	0.02	0.80	0.03	-0.2–0.3
P-total cholesterol	-0.14	0.096	-1.2	-2.7–0.2
P-HDL cholesterol	-0.02	0.76	- 0.6	-4.5–3.3
P-LDL cholesterol	-0.15	0.061	-1.5	-3.1–0.1
S-triglycerides	0.06	0.40	0.7	-0.9–2.3
B-leukocytes	0.12	0.10	0.7	-0.2–1.5
P-homocysteine	0.15	**0.033**	0.3	0.02–0.5

Bold indicates statistical significance of *P* values.

B, blood; CI, confidence interval; OGTT, oral glucose tolerance test; P, plasma; S, serum.

## Discussion

In this population-based study of relationships between glucose metabolism and abdominal aortic diameter and aneurysm in 65-year-old men, we could not confirm any previously reported negative relationships between DM, aneurysm development, and aortic diameter. On the contrary, both the prevalence of diabetes and HbA1c values were higher among men with AAA at ultrasound screening in unadjusted analysis, and furthermore adjusted analysis of s-insulin levels after OGTT confirmed relative hyperinsulinemia in men with AAA. Such associations have been previously shown. Hyperinsulinaemia and higher HOMA-IR were demonstrated in patients with AAA-diameter > 55 mm when compared to patients with smaller aneurysms [37], suggesting a relationship between aortic diameter and insulin resistance. Furthermore, the metabolic syndrome has also been retrospectively related to both larger AAA size and higher rupture rate [38]. Relationships between AAA and measures of obesity and insulin resistance are complex, however, an association between BMI and the presence of AAA but no relationship between BMI and AAA growth has been suggested [39]. Our study thus demonstrated that hyperinsulinemia as reflected by increase in s-insulin 2 hours after OGTT was related to both AAA presence and abdominal aortic diameter, independently of both WHR and smoking.

We found no differences between groups regarding SAF of AGE reflecting increased concentrations of glucose in dermal tissues and vessels. Previous evidence in this field is conflicting. Concentrations of the cross-linking AGE pentosidine have been negatively correlated with aortic diameter [40], suggesting a protective effect against aortic dilatation. On the other hand, increased SAF of AGE has been reported in patients with AAA compared with controls independently of the presence of concomitant atherosclerosis [41], and levels of the soluble receptor for AGE have been correlated to AAA size [42]. Blocking of this receptor has even been evaluated in animal models and proposed as a potential future treatment of AAA [43]. AGE has not previously been studied in subjects with relatively small screening-detected aneurysms, however, previous studies [36–38] have reported on patients with larger aneurysms.

Smoking and peripheral arterial disease were both more common among men with screening-detected AAA, as expected [44]. This group also showed lower total and LDL cholesterol levels despite adjustment for higher use of lipid-lowering agents [28,29] and higher p-homocysteine [45]. Furthermore, when all study subjects were assessed together, the association between p-homocysteine and aortic diameter [45,46] mediated by toxic effects on the endothelium, platelet activation and promotion of thrombosis [46] was corroborated.

On the other hand, we were not able to replicate findings of increased aortic c-f PWV compared to controls in patients with aneurysmal disease [25,47], reported in 18 patients with AAA with a mean diameter of 55 mm [25]. As aortic stiffness might increase locally in an aneurysmal segment [48], the discrepancy between our and previous [25] results might have been due to the smaller aneurysm size in the present study. Local measurements of stiffness [32] might have helped clarify this. Furthermore, the consequences for aortic elasticity in relation to abdominal [25] and thoracic [47] aneurysmal disease might differ substantially.

As AAA is 4–5 times more common in men than in women [44], screening for AAA is recommended [44] and conducted [49] in men only.

No conclusions can therefore be drawn regarding potential associations between impaired glucose metabolism, arterial stiffness, and abdominal aortic diameter in women. The low number of subjects studied constitutes another limitation. Furthermore, the studied sample might not be representative of the general population, as higher attendance to screening procedures is related to higher socioeconomic status [50]. Since both groups were recruited from the same screening program, however, this limitation applies to study subjects in both groups. A strength of this cross-sectional study on 65-year-old men is that there is no need for adjustment of age or gender in multivariate analysis. Furthermore, the three different measurements during the OGTT enable a more detailed analysis of the reported findings.

We did not longitudinally follow our patients for aneurysm growth in relationship to markers of glycaemia or obesity. Diabetes mellitus has been associated with lower growth rate of AAA [51], and it would have been relevant to assess the more sophisticated markers of glycaemia and stiffness evaluated in the present study in relation to AAA growth. As obesity is associated with prevalent AAA, but not with aneurysm growth [39], the influence of glucose metabolism upon formation and growth of AAA might be different. Insulin resistance and hyperinsulinaemia linked to obesity might promote AAA development, whereas hyperglycaemia and overt type 2 diabetes in later stages might counterbalance this effect. Such interplay between hyperinsulinaemia and hyperglycaemia has been described in relation to fracture-risk subjects from the same background population [52].

In conclusion, hyperinsulinemia might be related to both AAA and abdominal aortic diameter in 65-year-old men, whereas none of the previously reported negative associations between diabetes and AAA, or any relations between c-f PWV and AAA, could be confirmed. Whether glucose metabolism might be related to AAA growth can be assessed by continued follow-up of the study participants.

## Acknowledgements

The authors would like to thank Sophia Ågren-Witteschus, Cecilia Kennbäck, and her staff at the Clinical Research Unit, Skåne University Hospital, Malmö, Sweden for expert technical assistance.

Research funds at Skåne University Hospital, Region Skåne, the Hulda Ahlmroth Foundation, and from the Swedish Government under the LUA/ALF agreement.

### Conflicts of interest

There are no conflicts of interest.
